# *Brassica incana* Ten. (Brassicaceae): Phenolic Constituents, Antioxidant and Cytotoxic Properties of the Leaf and Flowering Top Extracts

**DOI:** 10.3390/molecules25061461

**Published:** 2020-03-24

**Authors:** Natalizia Miceli, Emilia Cavò, Monica Ragusa, Francesco Cacciola, Luigi Mondello, Laura Dugo, Rosaria Acquaviva, Giuseppe Antonio Malfa, Andreana Marino, Manuela D’Arrigo, Maria Fernanda Taviano

**Affiliations:** 1 Department of Chemical, Biological, Pharmaceutical and Environmental Sciences, University of Messina, Viale Palatucci, 98168 Messina, Italy; ecavo@unime.it (E.C.); lmondello@unime.it (L.M.); anmarino@unime.it (A.M.); mdarrigo@unime.it (M.D.); mtaviano@unime.it (M.F.T.); 2 Foundation “Prof. Antonio Imbesi”, University of Messina, Piazza Pugliatti 1, 98122 Messina, Italy; 3 Department of Experimental and Clinical Medicine, University “Magna Graecia” of Catanzaro, Viale Europa, Località Germaneto, 88100 Catanzaro, Italy; m.ragusa@unicz.it; 4 Department of Biomedical, Dental, Morphological and Functional Imaging Sciences, University of Messina, Via Consolare Valeria, 98125 Messina, Italy; cacciolaf@unime.it; 5 Unit of Food Science and Nutrition, Department of Medicine, University Campus Bio-Medico of Rome, via Àlvaro del Portillo 21, 00128 Rome, Italy; l.dugo@unicampus.it; 6 Chromaleont s.r.l., c/o Department of Chemical, Biological, Pharmaceutical and Environmental Sciences, University of Messina, Viale Palatucci, 98168 Messina, Italy; 7 BeSep s.r.l., c/o Department of Chemical, Biological, Pharmaceutical and Environmental Sciences, University of Messina, Viale Palatucci, 98168 Messina, Italy; 8 Department of Drug Science, Biochemistry Section, University of Catania, Viale Andrea Doria 6, 95123 Catania, Italy; racquavi@unict.it (R.A.); g.malfa@unict.it (G.A.M.)

**Keywords:** *Brassica incana* Ten., phenolic compounds, antioxidant activity, cytotoxicity, *Artemia salina* Leach

## Abstract

*Brassica incana* Ten. is an edible plant belonging to the Brassicaceae family. In this work, the phenolic composition and the antioxidant and cytotoxic properties of the hydroalcoholic extracts obtained from the leaves and the flowering tops of *B. incana* grown wild in Sicily (Italy) were studied for the first time. A total of 17 and 20 polyphenolic compounds were identified in the leaf and in the flowering top extracts, respectively, by HPLC-PDA-ESI-MS analysis. *Brassica incana* extracts showed in vitro antioxidant properties; the leaf extract displayed greater radical scavenging activity in the 2,2-diphenyl-1-picrylhydrazyl (DPPH) test than the flowering top extract (IC_50_ = 1.306 ± 0.049 mg/mL and 2.077 ± 0.011 mg/mL), which in turn had a stronger ferrous ion chelating ability than the other (IC_50_ = 0.232 ± 0.002 mg/mL and 1.147 ± 0.016 mg/mL). The cytotoxicity of the extracts against human colorectal adenocarcinoma (CaCo-2) and breast cancer (MCF-7) cell lines was evaluated through the 3-(4,5-dimethylthiazol-2-yl)-2,5-diphenyltetrazolium bromide (MTT) assay and the lactic dehydrogenase (LDH) release determination. The extracts showed cytotoxic efficacy against Caco-2 cells, with the flowering top extract being the most effective (about 90% activity at the highest concentration tested). In the brine shrimp lethality bioassay, the extracts exhibited no toxicity, indicating their potential safety.

## 1. Introduction

In recent decades, interest in new sources of health-promoting compounds has become a major research issue. Considerable attention has been paid to edible plants, especially those rich in bioactive phytochemicals. The Brassicaceae family includes 338 genera and 3709 species [1]. The genus *Brassica* is the most important one within the 51 genera and belongs to the subtribe Brassicinae, one of the nine subtribes of the Brassiceae tribe; the genus includes many species with economic and agricultural relevance [2,3]. Brassicaceae are recognized as rich sources of bioactive compounds, such as carotenoids, tocopherols, ascorbic acid, glucosinolates, and of phenolic compounds [4,5]. Strong epidemiological evidence demonstrated that these compounds may help to protect the human body against damage caused by reactive oxygen species and reduce the risk of chronic pathologies, including cardiovascular diseases and cancer [5].

In continuation of our earlier published studies on species belonging to the Brassicaceae family endemic to Sicily (Italy) [6,7,8], *Brassica incana* Ten. has been selected.

*Brassica incana*, a wild *B. oleracea*-related species, is a suffrutex growing up 100 cm high [3,9,10]. As reported in the Euro+Med PlantBase, *B. incana* is native to south-eastern Europe, including Albania, Bosnia-Herzegovina, Croatia, Greece, and Italy; the plant has also been introduced in Ukraine and Crimea [11]. In Italy, it grows in Tuscany, Lazio, Campania, Puglia, Basilicata, Calabria, and Sicily, where it mainly occurs on the cliffs and the calcareous rocky slopes, from sea level up to about 600-800 m of altitude [12,13].

*B. incana* is an edible plant [14]. Its use for the preparation of omelettes and of a typical Sicilian polenta, known as “Frascatula”, together with *Brassica fruticulosa* Cyr. and other wild herbs, is reported in Sicily.

Several species belonging to the *Brassica* genus have been the subject of numerous phytochemical investigations and studies on therapeutic potential in human and animal diseases [4,15,16,17]. Instead, concerning *B. incana*, very limited information is available. Indeed, to the best of our knowledge, only one article concerning the characterization of the volatile constituents of *B. incana* leaves and roots is present in the literature [18], whereas some research has been focused on the glucosinolates contained in leaves and seeds [19,20,21].

Based on the above considerations, the present work aimed to investigate the phenolic composition and certain biological properties of the hydroalcoholic extracts obtained from the leaves and the flowering tops of *B. incana* grown wild in Sicily (Italy). In particular, the study includes the quali–quantitative characterization of the phenolic constituents of the extracts by HPLC-PDA-ESI-MS analysis, and the evaluation of their antioxidant properties by in vitro assays and on *Escherichia coli*, which is used as biological substrate. The cytotoxic activity was assessed in both cell systems (human colorectal adenocarcinoma CaCo-2 and breast cancer MCF-7 cell lines) through the 3-(4,5-dimethylthiazol-2-yl)-2,5-diphenyltetrazolium bromide (MTT) assay and the lactic dehydrogenase (LDH) release determination and in vivo by the brine shrimp (*Artemia salina* Leach) lethality bioassay.

## 2. Results

### 2.1. Phytochemical Investigations

#### 2.1.1. Determination of Total Phenolic Content

The total phenolic content was found to be greater in the *B. incana* leaf extract than in the flowering top extract, with values of 37.20 ± 0.93 mg gallic acid equivalent (GAE)/g extract and 27.98 ± 0.32 mg GAE/g extract, respectively.

#### 2.1.2. Identification of Phenolic Compounds by HPLC-PDA-ESI-MS

**Figure 1:**
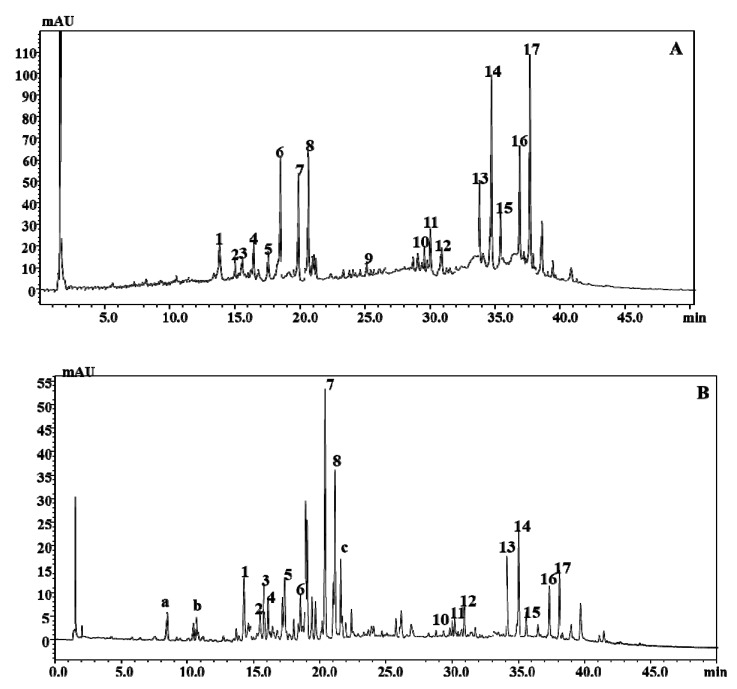
HPLC-PDA-ESI-MS polyphenolic fingerprint of *B. incana* leaf (**A**) and flowering top (**B**) hydroalcoholic extracts. Column: Ascentis Express C18, 15 cm × 4.6 mm, 2.7 μm d.p. (ESI, negative ionization mode). For peak identification, see Table 1.

The determination of the polyphenolic content of *B. incana* extracts was performed by HPLC-PDA-ESI-MS. Regarding the chromatographic analysis, a superficially porous C18 stationary phase at 1.0 mL/min was used, whereas as far as detection is concerned, both PDA and MS detection were employed. A total of 17 and 20 polyphenolic compounds were positively identified in the leaf and in the flowering top extracts, respectively. So far, no work has been carried on the characterization of the polyphenolic content in *B. incana*. The results illustrated in Figure 1 and Table 1 show that the extracts contain derivatives of the flavonols quercetin, kaempferol, and isorhamnetin, and of the hydroxycinnamic acids sinapic acid and ferulic acid, which were found in conjugation with sugars or hydroxycinnamic acids.

From a qualitative point of view, the polyphenolic profiles of the extracts are superimposable, except for the compounds kaempferol-3-*O*-diglucoside-7-*O*-glucoside, quercetin-3-sophoroside-7-glucoside, and feruloylmalate, which were identified exclusively in the flowering top extract.

Regarding quantification, since none of the compounds identified are commercially available, three selected reference standards were considered, namely quercetin-glucoside, kaempferol-glucoside, and isorhamentin-glucoside, for the determination of quercetin, kaempferol, and isorhamnetin derivates, respectively. In particular, for the leaf extract, isorhamnetin-3-glucoside-7-glucoside turned out to be the most abundant flavonoid (3.33 mg/g ± 0.54 % relative standard deviation (RSD)), followed by kaempferol-3-sinapoylsophoroside-7-glucoside (2.84 mg/g ± 0.77 %RSD) and kaempferol-3-feruloylsophoroside-7-glucoside (2.11 mg/g ± 0.98 %RSD); on the other hand, for the flowering top extract, kaempferol-3-feruloylsophoroside-7-glucoside (2.14 mg/g ± 0.48 %RSD) was the main flavonoid compound, followed by isorhamnetin-3-glucoside-7-glucoside (1.79 mg/g ± 0.32 %RSD) and quercetin-3-hydroxyferuloylsophoroside-7-glucoside (1.59 mg/g ± 0.57 %RSD).

### 2.2. Antioxidant Activity

**Figure 2:**
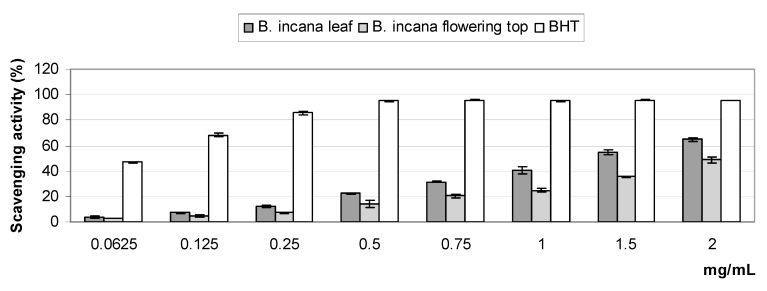
Free radical scavenging activity (2,2-diphenyl-1-picrylhydrazyl (DPPH) test) of *B. incana* leaf and flowering top hydroalcoholic extracts. Values are expressed as the mean ± SD (*n* = 3).

The results of the 2,2-diphenyl-1-picrylhydrazyl (DPPH) test are shown Figure 2. *Brassica incana* extracts exhibited radical scavenging activity, which increased with increasing amounts of the extracts. The leaf extract displayed higher activity than the flowering top extract, reaching about 65% and 50%, respectively, at the highest tested concentration. This was also confirmed by the IC_50_ values (1.306 ± 0.049 mg/mL and 2.077 ± 0.011 mg/mL, respectively). Compared to the standard BHT (IC_50_ = 0.065 ± 0.008 mg/mL), the activity of the extracts was moderate.

In the reducing power assay, the activity of both the extracts was found to be weak in comparison to that of the BHT.

**Figure 3:**
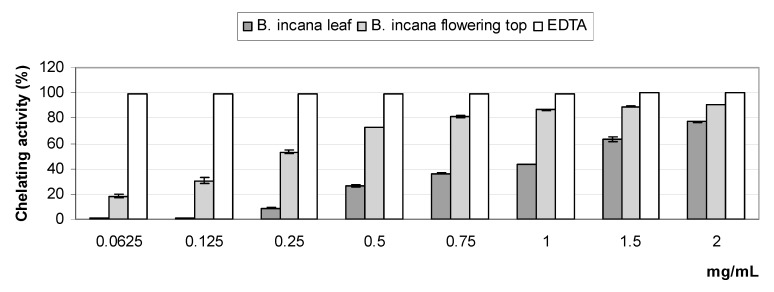
Ferrous ions chelating activity of *B. incana* leaf and flowering top hydroalcoholic extracts. Values are expressed as the mean ± SD (*n* = 3).

In the Fe^2+^ chelating activity assay, *B. incana* extracts exhibited relatively high and dose-dependent chelating properties (Figure 3). In this test, the flowering top extract was much more effective than the leaf one, reaching approximately 90% and 80% activity, respectively, at the highest tested concentration. The strongest efficacy of the flowering top extract was also confirmed by the calculated IC_50_ values (0.232 ± 0.002 mg/mL and 1.147 ± 0.016 mg/mL, respectively). However, the extracts were not as effective as the reference standard, EDTA (IC_50_ = 0.012 ± 3.546 × 10^−5^ mg/mL).

In the experimental model of oxidative stress induced by H_2_O_2_ in *E. coli*, the extracts did not show any protective effect on bacterial growth and survival.

### 2.3. Cytotoxic Activity

#### 2.3.1. Cell Viability Assay on Human Colorectal Adenocarcinoma (CaCo-2) and Breast Cancer (MCF-7) Cells

**Figure 4:**
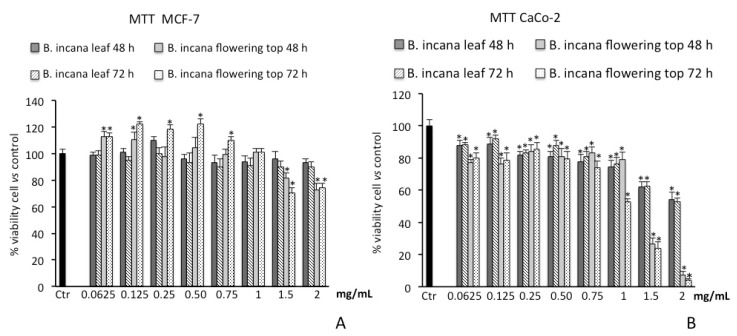
Cell viability in MCF-7 (**A**) and CaCo-2 (**B**) cells untreated and treated for 48 h and 72 h with *B. incana* leaf and flowering top hydroalcoholic extracts. Values are the mean ± SD of four experiments in triplicate. * Significant vs. untreated control cells: *p* < 0.001. MTT, 3-(4,5-dimethylthiazol-2-yl)-2,5-diphenyltetrazolium bromide.

We firstly verified that both extracts do not induce toxicity in human foreskin fibroblast (HFF-1) cells. For a time of 72 h at the highest tested concentration of 2 mg/mL, cell viability was not affected and was similar to untreated control cells. The results of the MTT bioassay on cancer cell lines showed that *B. incana* leaf extract was not able to reduce cell viability in MCF-7 at all concentrations tested at both 48 h and 72 h of exposure. Surprisingly, at the lowest dosages, a slight increase in cell viability was observed for the treatments at 48 h and 72 h for the flowering top extract. The flowering top extract exerted a significant cytotoxic effect on MCF-7 cell line, starting from the concentration of 1.5 mg/mL, with a reduction of viability of about 30% at 72 h of exposure (Figure 4A).

The treatment of CaCo-2 cells with different concentrations of *B. incana* extracts induced an inhibitory effect on succinate dehydrogenase activity at both 48 h and 72 h of exposure, resulting significant starting from 0.0625 mg/mL (Figure 4B). The flowering top extract was found to be the more effective, where the inhibitory effects reach a value of about 90% at the highest tested concentration (2 mg/mL).

The IC_50_ values for the cytotoxic activity of the flowering top extract on CaCo-2 cells are 1.25 ± 0.037 mg/mL and 1.1 ± 0.036 mg/mL at 48 h and 72 h, respectively.

**Figure 5:**
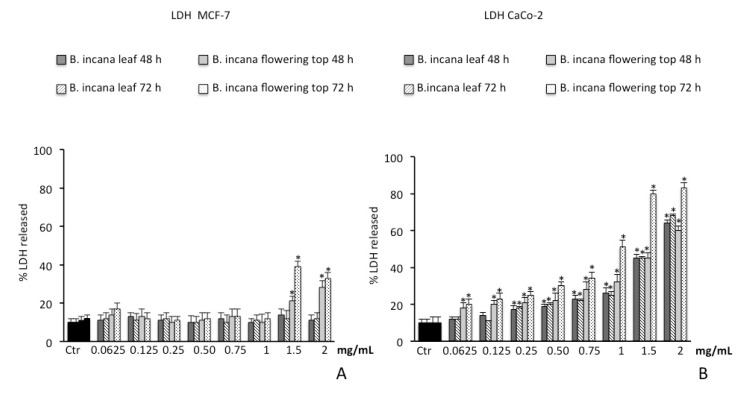
Lactic dehydrogenase (LDH) released in MCF-7 (**A**) and CaCo-2 (**B**) cells untreated and treated for 48 h and 72 h with *B. incana* leaf and flowering top hydroalcoholic extracts. Values are the mean ± SD of four experiments in triplicate. *Significant vs. untreated control cells: *p* < 0.001.

As shown in Figure 5A, consistent with the results obtained by MTT assay, after 48 h and 72 h of incubation with *B. incana* leaf extract, no appreciable LDH release was observed at all concentrations tested on MCF-7 cell line. A significant LDH release was spotted only for the *B. incana* flowering top extract at concentrations starting from 1.5 mg/mL.

Conversely, on CaCo-2 cell lines, a time- and dose-dependent LDH release was pointed out by the experimental model for both extracts. In particular, the necrotic effect is more evident for the *B. incana* flowering top extract after 72 h of exposure, being significant from the concentration of 0.0625 mg/mL (Figure 5B).

#### 2.3.2. Brine Shrimp Lethality Bioassay

The median lethal concentration values of *B. incana* extracts were found to be greater than 1000 μg/mL, indicating that they did not display any toxicity against brine shrimps.

## 3. Discussion

The presence of high amounts of phenolic compounds in *Brassica* spp. is well documented and it is recognized that the contribution of these secondary metabolites to the positive health effects of species belonging to this genus has generally been associated with their antioxidant capacity [4,22].

Herein, the phenolic profiles of *B. incana* leaf and flowering top hydroalcoholic extracts are characterized. The total phenolic content of the extracts, determined by the Folin–Ciocalteau method, was found to be higher than that previously reported for various hydroalcoholic extracts obtained from commonly consumed varieties of *Brassica oleracea*. Heimler et al. [23] evaluated the total phenolic content of 70% ethanol extracts obtained from edible parts of white cabbage (*B. oleracea* L. var. *capitata* L.), broccoli (*B. oleracea* L. conv. *botrytis* L. var. *italica* Plenk), Italian kale (*B. oleracea* L. var. *acephala* DC.), Savoy cabbage (*B. oleracea* L. var. *sabauda* L.), green cauliflower (*B. oleracea* L. conv. *botrytis* L. var. *botrytis* cv Verde di Macerata), cauliflower (*B. oleracea* L. conv. *botrytis* L. var. *botrytis* cv Snow ball), and Brussels sprouts (*B. oleracea* L. var. *gemmifera* Zencher), ranging from 4.30 to 13.80 mg GAE/g sample. A comparative study undertaken by Jaiswal et al. [24] to optimize the best solvents among 60% ethanol, acetone, and methanol for the extraction of polyphenols from *Brassica* vegetables showed that 60% methanolic extracts had the highest total phenolic content, which was 23.6, 20.4, and 18.7 mg GAE/g extract for broccoli, Brussels sprouts, and white cabbage, respectively.

HPLC-PDA-ESI-MS analysis showed that the extracts contain derivatives of the flavonols quercetin, kaempferol, and isorhamnetin, and of the hydroxycinnamic acids sinapic acid and ferulic acid, which are conjugated with sugar moieties or hydroxycinnamic acids. These types of compounds are very common in *Brassica* species [5,25,26,27,28,29,30]. Both flavonol glycosides and hydroxycinnamic esters have been reported to possess antioxidant activity [31,32].

Flavonoids and phenolic acids can act as hydrogen or electron donors, reducing agents, and metal ion chelators resulting from different conjugations and varying numbers of hydroxyl groups [33]. Thus, the antioxidant potential of *B. incana* extracts was investigated by means of in vitro assays based on these different mechanisms. The primary (chain-breaking) antioxidant properties were examined using two different tests: the DPPH test, which is based on a combination of hydrogen atom transfer (HAT) and single electron transfer (SET) reactions, and the reducing power assay, which is a SET-based method [34,35,36]. The secondary (preventive) antioxidant ability was determined by the Fe^2+^ chelating activity assay.

The results of the in vitro antioxidant tests highlighted that *B. incana* leaf and flowering top extracts have antioxidant properties; the former displays greater radical scavenging activity than the latter, which in turn has a stronger chelating ability.

In the reducing power assay, both the extracts were found to possess weak activity in the range of concentrations tested (0.0625–2 mg/mL). The in vitro antioxidant activities of the main *Brassica* crops have been studied by different authors; similarly to our results, some of these investigations have highlighted good radical scavenging activity and low reducing power in *Brassica* spp. extracts [37,38].

The observed radical scavenging properties could be mainly attributed to the phenolic compounds detected in the extracts. A few studies aimed at establishing the antioxidant properties of phenolic compounds isolated from *Brassica* species have been previously reported. Some authors have investigated the contribution of flavonoid glycosides and hydroxycinnamic acid derivatives to the antioxidant activity of *Brassica oleracea* var. *sabellica* (kale), by evaluating their ability to scavenge the ABTS radical in order to determine the structure–antioxidant-activity relationships [26,27]. In the work of Zietz and colleagues [26], the quercetin derivatives monoacylated with sinapic, hydroxyferulic, ferulic, and caffeic acids; along with those glycosidated with sophorose in position 3-*O* and glucose or diglucose in position 7-*O*, were found to display higher radical scavenging activity than the kaempferol ones, together with the hydroxycinnamic acid ester 1,2-disinapoyl-gentiobiose. Several research studies on the antioxidant potential of plant extracts highlighted a strong correlation between ABTS and DPPH assays [39,40,41]. Thus, it can be hypothesized that quercetin derivatives with the same structural features and 1,2-disinapoyl-gentiobiose contained in *B. incana* extracts are the main derivatives responsible for the observed free radical scavenging activity.

In a study carried out by Yokozawa et al. [42], isorhamnetin diglucoside isolated from the leaves of *Brassica juncea* (L.) Czern. was found to be inactive in the DPPH test. Therefore, this compound, which represents the main flavonoid detected in *B. incana* leaf extract, would not be involved in the scavenging effect.

Concerning the chelating properties of *B. incana* extracts, the activity of the flowering top extract was found to be about five time stronger than that of the leaf one, despite the lower phenolic content. This suggests that the phenolic compounds are only partially responsible for the observed chelating activity, and also that other polar phytochemicals contained in the extracts may contribute to this activity.

The cancer preventive properties of various *Brassica* species have been reported. A regular intake of vegetables such as broccoli, cabbage, and cauliflower is well-known to reduce the risk of cancer [4,43,44]. Although this effect has been mainly related to the glucosinolate compounds and their derivative products, flavonoids and other phenolics also contribute to it [5,15,16].

The cytotoxicity of *B. incana* extracts in MCF-7 and CaCo-2 cell lines was evaluated by the MTT assay and by determination of LDH release as an index of necrotic death. Necrosis is a type of cell death that is morphologically characterized by swelling and rupture of intracellular organelles, leading to the disruption of the plasma membrane and the release of intracellular contents. LDH is a soluble cytoplasmic enzyme that is present in almost all cells and is released into extracellular space due to the loss of membrane integrity in dying cells. Thus, the determination of LDH release can be utilized as a useful method for detection of necrosis, and therefore as a measure of cytotoxicity [45,46]. The capacity of a plant extract to induce necrotic cell death could potentially be a valuable adjunctive therapy in cancer treatment [47].

*Brassica incana* leaf and flowering top extracts showed different cytotoxic effects on the investigated cell lines. Both extracts showed no appreciable cytotoxic activity on MCF-7 cell line; only the flowering top extract showed a slight inhibitory action at higher dosages after 72 h of treatment, accompanied by necrotic cell death. This result is possibly due to the high drug resistance that MCF-7 breast cancer cells possess [48]. Conversely, both the extracts showed a cytotoxic effect in a dose dependent manner, decreasing cell viability of CaCo-2 cells. The different response of CaCo-2 cells in an in vitro model of colon cancer is probably related to the strong absorption capacity of this cell line, making it more sensitive to cytotoxic actions of the extracts [49].

The results highlighted that the flowering top extract exerted a higher cytotoxic action than the leaf one, despite the lower phenolic content and radical scavenging activity. The greater activity of the flowering top extract may depend on its strong ferrous ions chelating properties. Indeed, it has been shown that some metals, such as copper and iron, play a significant role in the rapid proliferation of cancer cells [50].

Further research should be carried out to better clarify the underlying mechanism responsible for the observed cytotoxic effect.

The cytotoxicity of the extracts was also assessed by the brine shrimp lethality bioassay. It represents a simple and low-cost technique for predicting the toxicity of plant extracts in order to consider their safety. It is also a useful system for testing plant extract bioactivity, which in most cases correlates reasonably well with cytotoxic and anti-tumor properties [51]. However, various studies did not highlight this relationship [6,52,53]. Despite the observed activity against tumor cell lines, in this experimental model *B. incana* extracts exhibited no toxicity against brine shrimp larvae, which indicated their potential safety.

## 4. Materials and Methods

### 4.1. Chemicals

LC-MS-grade acetonitrile (ACN) and water (H_2_O), kaempferol-3-*O*-glucoside, isorhamnetin-3-*O*-glucoside, and quercetin-3-*O*-glucopyranoside were obtained from Merck Life Science (Merck KGaA, Darmstadt, Germany). Methanol (MeOH) was purchased from Baker Analyzed Reagents. Ferrous chloride (FeCl_2_) was obtained from Carlo Erba (Milan, Italy). Luria–Bertani (LB) broth medium was supplied from Oxoid (Basingstoke, UK). Unless indicated otherwise, all chemicals were purchased from Sigma-Aldrich (Milan, Italy).

### 4.2. Plant Material and Extraction Procedure

*Brassica incana* Ten. was collected around Capo d’Orlando (Messina, Italy), with the leaves collected in November 2018 and the flowering tops in May 2019. The taxonomic identification was confirmed by Prof. S. Ragusa, Department of Health Sciences, University Magna Graecia of Catanzaro (Italy). A voucher specimen (1108/18) was deposited in the same department.

After harvesting, the plant material was washed, blended, frozen, and then lyophilized. Subsequently, it was subjected to a preventive maceration at 25 °C with 70% MeOH for an hour. The extraction was carried out with 70% MeOH in an ultrasonic bath at 50 °C for 15 min, repeated four times. The filtrates were combined and evaporated to dryness by rotavapor; the yields of the leaf and flowering top extracts, compared to 100 g of lyophilized plant material, were 26.47% and 33.16%, respectively.

### 4.3. Phytochemical Investigations

#### 4.3.1. Determination of Total Phenolic Content

The total phenolic contents of *B. incana* extracts were determined by the Folin–Ciocalteau method compared to the calibration curve of gallic acid phenol compound used as a standard [54]. The extracts were dissolved in 70% MeOH; to 100 µL of each sample solution, 0.2 mL Folin–Ciocalteu reagent, 2 mL of H_2_O, and 1 mL of 15% Na_2_CO_3_ were added. After 2 h incubation at room temperature, the absorbance was measured at 765 nm with a UV-1601 spectrophotometer (Shimadzu, Milan, Italy). The total polyphenols were estimated as gallic acid equivalent (GAE) and expressed in mg GAE/g extract (dw) ± standard deviation (SD). The data were obtained from the average of three independent determinations.

#### 4.3.2. Identification of Phenolic Compounds by HPLC-PDA-ESI-MS

Instrumentation: The analyses were carried out using a Shimadzu HPLC system (Kyoto, Japan) equipped with a CBM-20A controller, two LC-20AD pumps, a DGU-20A3 degasser, a SIL-20AC autosampler, a SPD-M20A photo diode array detector (PDA), and a triple quad mass analyzer (LCMS-8050, Shimadzu, Kyoto Japan) equipped with an ESI interface in negative ionization mode. Data acquisition was performed by Shimadzu LabSolution software version 5.65 (Kyoto, Japan).

Samples Preparation: Ten milligrams of *B. incana* leaf or flowering top extracts was dissolved in 1 mL of MeOH.

Chromatographic conditions: Analyses were performed on a Ascentis Express C18, 15 cm × 4.6 mm internal diameter (I.D.), with a particle size of 2.7 μm (Merck Life Science, Merck KGaA, Darmstadt, Germany). The mobile phase was composed of water/formic acid (99.9:0.1) (solvent A) and ACN/formic acid (99.9:0.1) (solvent B), with the following gradient: 0 min, 0% B; 5 min, 5% B; 15 min, 10% B; 30 min, 20% B; 60 min, 50% B; 70 min, 100% B; 71 min, 0% B. The injection volume was 10 μL. The flow rate was 1 mL/min and it was split to 0.2 mL/min prior to MS detection.

PDA conditions: The wavelength range was 200–400 nm and the chromatograms were extracted at 280 nm. The time constant was 25 ms and the sample frequency was 40 Hz.

MS conditions: The MS acquisition was performed using ESI in negative mode under the following conditions: mass spectral range: 100–1400 *m*/*z*; scan speed: 2727 u/sec; event time: 0.5 sec; nebulizing gas (N_2_) flow: 3 L/min; interface temperature: 300 °C Heat block: 400 °C, desolvation line (DL) temperature: 250 °C; DL voltage −34 V; probe voltage 4.5 kV; array voltage: 1.0 V, RF voltage: 90 V; detection gain 1.0 kV. Construction of calibration curves: In absence of the reference materials for the quantification of the polyphenolic content, three standards were selected, which were representative of the chemical classes under study, namely kaempferol-3-*O*-glucoside (y = 17660x − 10681, R² = 0.9963, limit of detection (LOD) = 0.023, limit of quantification (LOQ) = 0.072), isorhamnetin-3-*O*-glucoside (y = 14948x − 2966.9, LOD = 0.032, LOQ = 0.098), and quercetin-3-*O*-glucopyranoside (y = 13424x + 898.59, R² = 0.9939, LOD = 0.013, LOQ = 0.043). Standard calibration curves were prepared in a concentration range of 0.1-100 mg/L, considering five different concentration levels. Triplicate injections were made for each level, and a linear regression was generated. The calibration curves with the external standards were obtained using their concentrations (mg/L) with respect to the area obtained from the integration of the PDA peaks at a wavelength of 330 nm. The amount of the compound was finally expressed in mg/g of extract.

### 4.4. Antioxidant Activity

#### 4.4.1. Free Radical Scavenging Activity

The free radical scavenging activity of *B. incana* extracts was determined using the 2,2-diphenyl-1-picrylhydrazyl (DPPH) test [54]. Different amounts of each extract were dissolved in 70% MeOH to obtain final concentrations in the range of 0.0625–2 mg/mL. A volume of 0.5 mL of each sample was mixed with 3 mL of daily prepared methanol DPPH solution (0.1 mM) and incubated for 20 min at room temperature in the dark. Then, the optical density change at 517 nm was measured with a model UV-1601 spectrophotometer (Shimadzu). Butylated hydroxytoluene (BHT) was used as a reference compound. The scavenging activity was measured as the decrease in absorbance of the samples versus DPPH standard solution. The results were obtained from the average of three independent experiments, and are reported as mean radical scavenging activity percentage (%) ± SD and mean 50% inhibitory concentration (IC_50_) ± SD.

#### 4.4.2. Measurement of Reducing Power

The reducing power of *B. incana* extracts was evaluated by Fe^3+^-Fe^2+^ transformation method [54]. Different amounts of each extract were dissolved in 70% MeOH to obtain final concentrations in the range of 0.0625–2 mg/mL. A volume of 1 mL of each sample was mixed with 2.5 mL of phosphate buffer (0.2 M, pH 6.6) and 2.5 mL of 1% potassium ferrycyanide [K_3_Fe(CN)_6_]. The mixture was incubated at 50 °C for 20 min, then it was cooled rapidly, mixed with 2.5 mL of 10% trichloroacetic acid, and centrifuged at 3000 rpm for 10 min. The resulting supernatant (2.5 mL) was mixed with 2.5 mL of distilled water and 0.5 mL of 0.1% fresh ferric chloride (FeCl_3_), and the absorbance was measured at 700 nm after 10 min of incubation at room temperature in the dark; the increased absorbance of the reaction mixture indicates an increase in reducing power. As blank, an equal volume (1 mL) of water was mixed with a solution prepared as described above. Ascorbic acid and BHT were used as reference. The results were obtained from the average of three independent experiments, and are expressed as mean absorbance values ± SD and ascorbic acid equivalent (ASE/mL) ± SD.

#### 4.4.3. Ferrous Ion (Fe^2+^) Chelating Activity

The Fe^2+^ chelating activity of *B. incana* extracts was estimated by measuring the formation of the Fe^2+^-ferrozine complex [54]. Different amounts of each extract were dissolved in 70% MeOH to obtain final concentrations in the range of 0.0625–2 mg/mL. A volume of 1 mL of each sample was mixed with 0.5 mL of MeOH and 0.05 mL of 2 mM FeCl_2_. Then, 0.1 mL of 5 mM ferrozine was added to initiate the reaction; the mixture was shaken vigorously and incubated at room temperature in the dark for 10 min. The absorbance of the solution was measured spectrophotometrically at 562 nm. The control contained FeCl_2_ and ferrozine, which are complex formation molecules. Ethylenediaminetetraacetic acid (EDTA) was used as the reference standard The results were obtained from the average of three independent experiments and are reported as mean inhibition of the ferrozine-(Fe^2+^) complex formation (%) ± SD and IC_50_ ± SD.

#### 4.4.4. Protective Effect on *Escherichia coli* Growth and Survival under Peroxide Stress

The protective effects of *B. incana* extracts on bacterial growth and survival from the oxidative stress induced by hydrogen peroxide (H_2_O_2_) were evaluated in *Escherichia coli* ATCC 25922 [54]. The strain was obtained from the Department of Chemical, Biological, Pharmaceutical and Environmental Sciences, University of Messina, with in-house culture collection (Messina, Italy). After overnight growth in LB medium, the bacterial suspension was centrifuged (10 min, 3500 rpm), resuspended in LB fresh medium to obtain a final optical density at 600 nm (OD_600_) = 0.1, and then grown aerobically at 37 °C under low shaking (150 rpm). When the growth reached the mid-log phase (OD_600_ = 0.6), the bacterial suspension was centrifuged and the OD_600_ adjusted to 0.2 value with fresh medium and aliquoted, then *B. incana* extracts (1 mg/mL) and the reference standard quercetin (0.2 mM) were added. To establish the protective effect of the extracts against *E. coli* growth inhibition induced from oxidative stress, when OD_600_ was equal to 0.4, bacteria were treated with 2 mM H_2_O_2_, and the growth was monitored every 20 min for 180 min.

For survival studies, the bacteria (OD_600_ = 0.4) were exposed to 10 mM H_2_O_2_ for 30 min, then an aliquot of each sample was diluted in 0.9% NaCl to obtain serial dilutions (1:10). Each sample was poured onto LB agar plates, and the cell survival was estimated after 24 h of incubation at 37 °C by counting the number of viable colonies.

The results were obtained from the average of three independent experiments and expressed as mean absorbance ± SD and survival (%) ± SD for protective effect on *E. coli* growth and survival, respectively.

### 4.5. Cytotoxic Activity

#### 4.5.1. Cell viability Assay on Human Colorectal Adenocarcinoma (CaCo-2) and Breast Cancer (MCF-7) Cells

Cell culture and treatments: Human colorectal carcinoma cells (CaCo-2), obtained from the American Type Culture Collection (Rockville, MD, USA), were cultured in Dulbecco’s modified Eagle’s medium (Gibco BRL, Life Technologies, Grand Island, NY, USA) supplemented with 10% fetal calf serum, 1 mmol/L sodium pyruvate, 2 mmol/L L-glutamine, streptomycin (50 mg/mL), and penicillin (50 U/mL) in 5% CO_2_ at 37 °C. MCF-7 breast cancer cells (ATCC cell bank, Rockville, MD, USA) were cultured in Roswell Park Memorial Institute (RPMI) medium containing 10% fetal bovine serum (FBS), 100 U/mL penicillin, and 100 μg/mL streptomycin in 5% CO_2_ at 37 °C. Human foreskin fibroblast (HFF-1, ATCCVR SCRC-1041TM) cells were cultured in Dulbecco’s modified Eagle’s medium, 4.5 g/L glucose, and penicillin or treptomycin (100 U/mL penicillin and 100 μg/mL streptomycin), with 15% fetal bovine serum.

HFF-1 cell line was exposed for 72 h at the highest concentration of 2 mg/mL of both extracts, while CaCo-2 and MCF-7 cell lines were exposed to the different concentrations of *B. incana* leaf and flowering top extracts for 48 h and 72 h. Extracts were dissolved in medium to the final concentrations, ranging from 0.0625 to 2 mg/mL of extract.

MTT bioassay: Cell viability was assessed by MTT assay on a 96-multiwell plate (8 × 10^3^ cells/well), as previously described [55]. Inside the metabolically active cells, the tetrazolium salt is converted to yield-colored formazan. The amount of formazan is proportional to the number of living cells. The optical density was measured with a microplate spectrophotometer reader (Titertek Multiskan, Flow Laboratories, Helsinki, Finland) at λ = 570 nm. Results are expressed as percentage of cell viability with respect to control (untreated cells).

Lactic dehydrogenase release: The presence of LDH in a medium of cultured cells is a useful tool to evaluate cell necrosis as a result of cell membrane disruption. LDH activity was measured spectrophotometrically in the culture medium and in the cellular lysates at λ = 340 nm by measuring β-Nicotinamide-adenine dinucleotide (NADH) reduction [56]. LDH release was calculated as the percentage of the total amount, considered as the sum of the enzymatic activity present in the cellular lysate and in the culture medium. Results are expressed as percentage of LDH released.

One-way analysis of variance (ANOVA) followed by Bonferroni’s *t*-test was performed in order to estimate significant differences among groups. Data were reported as mean values ± SD and differences among groups were considered to be significant at *p* < 0.001.

#### 4.5.2. Brine Shrimp Lethality Bioassay

The toxic potential of *B. incana* extracts was investigated in brine shrimp (*Artemia salina* Leach) [54]. Ten brine shrimp larvae, taken 48 h after initiation of hatching in artificial seawater, were transferred to each sample vial, then artificial seawater was added to obtain a final volume of 5 mL. Different concentrations of each extract were added (10–1000 µg/mL) and the brine shrimp larvae were incubated for 24 h at 25–28 °C. Then, the surviving larvae were counted using a magnifying glass. The assay was carried out in triplicate, and median lethal concentration (LC_50_) values were determined by the Litchfield and Wilcoxon’s method. Extracts giving LC_50_ values greater than 1000 μg/mL were considered non-toxic.

## 5. Conclusions

In the present work, the polyphenolic profile and the antioxidant and cytotoxic properties of *B. incana* leaves and flowering tops are reported for the first time.

Based on the data obtained from the different in vitro tests carried out to establish the antioxidant potential of the *B. incana* hydroalcoholic extracts, it can be stated that they have stronger secondary antioxidant properties than the primary ones. The chelating activity of the extract obtained from the flowering tops is particularly relevant.

*Brassica incana* extracts showed cytotoxic action against Caco-2 cells, probably through the activation of some biochemical pathways related to the necrotic cell death, and the flowering top extract was the most effective.

Overall, the present findings highlighted that *Brassica incana* represents a safe source of bioactive compounds, providing a valuable contribution to the knowledge of the phytochemical composition and the biological properties of this edible plant.

## Figures and Tables

**Table 1:** Polyphenolic determination of *B. incana* leaf (A) and flowering top (B) hydroalcoholic extracts by LC-PDA-MS/MS.

Peak	Compound	t_R_ (min)	[M − H]^−^	mg/g ± %RSD (A)	mg/g ± %RSD (B)
a	Kaempferol-3-*O*-diglucoside-7-*O*-glucoside	8.4	773 (609)	-	1.11 ± 1.44
b	Quercetin-3-sophoroside-7-glucoside	10.7	787 (625)	-	1.32 ± 1.32
1	Quercetin-3-hydroxyferuloylsophoroside-7-glucoside	13.9	979 (625)	1.91 ± 0.52	1.59 ± 0.57
2	Quercetin-3-caffeoylsophoroside-7-glucoside	15.2	949 (625)	1.55 ± 1.23	1.22 ± 1.41
3	Kaempferol-3-hydroxyferuloylsophoroside-7-glucoside	15.6	963 (801)	1.43 ± 1.21	1.41 ± 1.39
4	Quercetin-3-sinapoyltriglucoside-7-glucoside	16.3	1155 (831)	1.22 ± 1.11	0.62 ± 1.87
5	Quercetin-3-feruloyl-diglucoside-7-glucoside	17.6	963 (801)	1.52± 1.32	1.17 ± 1.11
6	Kaempferol-3-sinapoylsophoroside-7-glucoside	18.5	977 (817)	2.84 ± 1.52	0.59 ± 1.98
7	Kaempferol-3-feruloylsophoroside-7-glucoside	19.9	947 (609)	2.11 ± 0.98	2.14 ± 0.48
8	Isorhamnetin-3-glucoside-7-glucoside	20.7	639 (747)	3.33 ± 0.77	1.79 ± 0.32
c	Feruloylmalate	21.3	309	-	N.Q.
9	Sinapoylmalic acid	25.2	339 (223)	N.Q.	N.Q.
10	Sinapoyl-hydroxyferuloyldiglycoside	28.6	739 (515)	N.Q.	N.Q.
11	Isorhamnetinglycoside	29.8	477 (315)	1.28 ± 0.54	0.57 ± 2.01
12	Kaempferolglycoside	30.7	447 (285)	0.64 ± 1.08	0.42 ± 1.94
13	Disinapoylgentiobiose	33.6	753 (529)	N.Q.	N.Q.
14	Sinapoylferuloylgentiobiose	34.8	723 (529)	N.Q.	N.Q.
15	Diferuloyldiglucoside	35.4	693 (499)	N.Q.	N.Q.
16	Trisinapoylgentiobiose	36.7	959 (735, 529)	N.Q.	N.Q.
17	Feruloyldisinapoylgentiobiose	37.7	929 (705, 511)	N.Q.	N.Q.

N.Q.: Not quantified.
